# Prediction of presence of kidney disease in patients undergoing intravenous iodinated contrast enhanced computed tomography: a validation study

**DOI:** 10.1007/s00330-016-4478-0

**Published:** 2016-07-19

**Authors:** Sanne M. Schreuder, Jaap Stoker, Shandra Bipat

**Affiliations:** Department of Radiology, Academic Medical Centre, G1-212, University of Amsterdam, Meibergdreef 9, 1105 AZ Amsterdam, The Netherlands

**Keywords:** Acute kidney injury, Kidney disease, Computed tomography, Contrast medium, Risk factors

## Abstract

**Objectives:**

To validate two previously presented models containing risk factors to identify patients with estimated glomerular filtration rate (eGFR) <60 ml/min/1.73 m^2^ or eGFR <45 ml/min/1.73 m^2^.

**Methods:**

In random patients undergoing intravenous contrast-enhanced computed tomography (CECT) the following risk factors were assessed: history of urological/nephrological disease, hypertension, diabetes mellitus, anaemia, congestive heart failure, other cardiovascular disease or multiple myeloma or Waldenström disease. Data on kidney function, age, gender and type and indication of CECT were also registered. We studied two models: model A—diabetes mellitus, history of urological/nephrological disease, cardiovascular disease, hypertension; model B—diabetes mellitus, history of urological/nephrological disease, age >75 years and congestive heart failure. For each model, associations with eGFR <60 ml/min/1.73 m^2^ or eGFR <45 ml/min/1.73 m^2^ was studied.

**Results:**

A total of 1,001 patients, mean age 60.36 years were included. In total, 92 (9.2 %) patients had an eGFR <60 ml/min/1.73 m^2^ and 11 (1.1 %) patients an eGFR <45 ml/min/1.73 m^2^. Model A detected 543 patients: 81 with eGFR <60 ml/min/1.73 m^2^ (missing 11) and all 11 with eGFR <45 ml/min/1.73 m^2^. Model B detected 420 patients: 70 (missing 22) with eGFR <60 ml/min/1.73 m^2^ and all 11 with eGFR <45 ml/min/1.73 m^2^. Associations were significant (*p* < 0.05).

**Conclusions:**

Model B resulted in the lowest superfluous eGFR measurements while detecting all patients with eGFR <45 ml/min/1.73 m^2^ and nearly all with eGFR <60 ml/min/1.73 m^2^.

***Key Points*:**

• *Less than 10% of patients undergoing contrast-enhanced CT have an eGFR of <60ml/min/1.73m* ^*2*^

• *Four risk factors can be used to detect pre-existent kidney disease*

• *It is safe to reduce eGFR measurements using a four-risk-factor model*

**Electronic supplementary material:**

The online version of this article (doi:10.1007/s00330-016-4478-0) contains supplementary material, which is available to authorized users.

## Introduction

The use of computed tomography (CT) with intravenous iodinated contrast enhancement has increased over recent decades [1,2]. Contrast-induced nephropathy (CIN) is a major adverse effect of intravascular administration of iodinated contrast medium [3]. In most studies CIN is defined as an absolute (≥0.5 mg/dL) or relative (≥25 %) increase in serum creatinine within 48–72 h after iodinated contrast medium administration in the absence of another explanation for the rise in serum creatinine [4–12]. To prevent CIN, several guidelines have been introduced over the years, which are based on identifying patients at risk of CIN prior to intravenous iodinated contrast medium administration and treatment of these patients.

Pre-existent kidney disease is considered the most important risk for CIN [13–21]. In general two different cut-off values for pre-existent kidney disease are described in guidelines: stage 3A or higher [estimated glomerular filtration rate (eGFR) <60 ml/min/1.73 m^2^] and stage 3B or higher (eGFR <45 ml/min/1.73 m^2^) [13–21]. If pre-existent kidney disease is detected, other risk factors are assessed to determine whether precautions should be taken before intravenous iodinated contrast administration. The other risk factors stated in the literature are age (>60 or >75 years), hypertension, diabetes mellitus, use of nephrotoxic medication, urological or nephrological history, (cardio)vascular disease, congestive heart failure, anaemia, active malignancy and multiple myeloma or morbus Waldenström [13–23].

Since CIN prevention guidelines are quite extensive and demanding for patients, physicians and the healthcare system in general, it is questionable if these guidelines are suitable for the problem at hand. Firstly, the most mentioned comment is that CIN prevention guidelines are mainly based on patients undergoing coronary angiography studies [18–20]. Secondly, the eGFR cut-off value of <60 ml/min/1.73 m^2^ is also an important topic of debate and is increasingly considered as too strict for a CIN prevention strategy in patients receiving intravenous contrast-enhanced computed tomography (IV CECT). Two European guidelines recently redefined their risk profile for CIN in patients receiving intravenous iodinated contrast medium [15,21]. Patients at risk for CIN were defined as patients with pre-existent eGFR <40 ml/min/1.73 m^2^ or <45 ml/min/1.73 m^2^ in combination with other risk factors such as diabetes mellitus and advanced age [15,21]. The option of lowering the eGFR cut-off value is also mentioned in the Canadian Association of Radiologists and the American College of Radiology CIN prevention guidelines [19,20]. Thirdly, the most commonly used approach to identify those at risk for CIN is to first determine eGFR in all patients before iodinated contrast medium administration and subsequently identify other risk factors [24]. This leads to superfluous eGFR measurements and therefore some international guidelines indicate that other risk factors should be assessed first and that eGFR should only be determined in those patients considered at risk of CIN [17–21]. This may reduce costs and is less cumbersome for the patient.

An earlier systematic review presented risk models for predicting chronic kidney disease (CKD). Most of the prediction models were applied in a heterogeneous population. The authors concluded that the risk models had a modest-to-acceptable discriminatory performance, but would need to be better calibrated and externally validated [25].

Another study reported the associations between eGFR (eGFR <45 ml/min/1.73 m^2^ and eGFR <60 ml/min/1.73 m^2^) and different risk factors in patients undergoing IV CECT [26]. The results of that study showed that screening for all relevant risk factors for kidney disease is less accurate than models containing only four risk factors. By using a model including diabetes mellitus, history of urological/nephrological disease, cardiovascular disease (all cardiovascular disease including congestive heart failure and peripheral arterial disease) and hypertension, all patients with an eGFR <45 ml/min/1.73 m^2^ were detected. Only 12 patients with an eGFR <60 ml/min/1.73 m^2^ were missed, of which nine patients had no other risk factors and were not considered as patients at risk for CIN [26]. Another model containing diabetes mellitus, history of urological/nephrological disease, age >75 years and congestive heart failure resulted in a further reduction in superfluous eGFR measurements. By using this model, all patients with an eGFR <45 ml/min/1.73 m^2^ were detected. However more patients (26) with an eGFR <60 ml/min/1.73 m^2^ were missed, compared with the above-mentioned model. Of these 26 patients, 16 had two risk factors and were considered as patients at risk of CIN [26].

This previous study reported no validation of the proposed models and therefore the aim of this study was to validate those models in a comparable study population of patients undergoing IV CECT.

## Materials and methods

### Study settings

We conducted a prospective cohort study in a university hospital between 15 July 2014 and 1 September 2015. Requirement for informed consent was waived by the medical ethics committee since our study did not interfere with standard care and patient burden was considered minimal.

### Patient population

We included patients who were scheduled to undergo IV CECT. We randomly asked patients at the desk to complete a questionnaire (Appendix 1). Patients aged <18 years, patients who were admitted to the emergency department or the intensive care unit were excluded because most guidelines do not apply to these patient groups. In our institute we use two types of contrast medium for IV CECT: Iopromide (Ultravist 300; Bayer, Leverkusen, Germany) or Iomeprol (Iomeron 400; Bracco, Milan, Italy). Both are low-osmolar and non-ionic contrast agents.

### Data collection

#### Risk factors

We assessed the presence of risk factors associated with kidney disease (decreased eGFR) that were mentioned in recent literature and most CIN prevention guidelines [17–19,21–23,27]. This was done by asking the patients to complete a questionnaire on the day of the IV CECT. Patients were asked whether they had a history of urological/nephrological disease or whether they suffered from hypertension, diabetes mellitus, anaemia, congestive heart failure, other cardiovascular disease (such as peripheral arterial disease, stroke, etc.) or multiple myeloma or Waldenström disease. We considered these risk factors present if the patient was diagnosed and/or treated for these risk factors regardless of the effect of the treatment. We did not categorize these risk factors according to severity or classifications.

#### Kidney function

From the electronic patient records we collected information on kidney function (eGFR and serum creatinine) before the intravenous iodinated contrast medium administration. The eGFR was determined using the four point Modification of Diet in Renal Disease (MDRD) formula which takes into account age, race and sex [28,29]. We also documented the time between the eGFR measurement and the intravenous iodinated contrast-enhanced examination. The eGFR was known in all patients as indicated by the national guideline used in our hospital. This means that eGFR was measured <3 months prior to the IV CECT in patients with known or suspected kidney disease or any risk factors. In all other patients eGFR was measured <12 months prior to IV CECT, following these national guidelines [16,18]. We also registered characteristics such as age, race (Afro-European), gender and the indication for the IV CECT.

### Models

We selected two models from a previous study [26] that were best in identifying patients with pre-existent kidney disease, models 3 and 4, and we renamed them to model A and B, respectively. These models were created in a previous study based on interim analysis [26]. All models contained diabetes mellitus because diabetes in combination with pre-existent kidney disease (decreased eGFR) are considered to be major predictors for CIN regardless of the presence of other risk factors [13–21]. The other risk factors were chosen because they were easy to objectify, thereby minimising interpretation variability. All of these risk factors were mentioned in current guidelines and literature on CIN prevention [13–23,27].

Model A includes diabetes mellitus, history of urological/nephrological disease, cardiovascular disease (all cardiovascular disease including congestive heart failure and peripheral arterial disease) and hypertension. Model B includes diabetes mellitus, history of urological/nephrological disease, age >75 years and congestive heart failure. If patients had one or more risk factors for kidney disease that were incorporated in these models, measurement of eGFR was indicated for detection of kidney disease, prior to intravenous contrast medium administration.

Patients that had an eGFR <60 ml/min/1.73 m^2^ or <45 ml/min/1.73 m^2^, who were detected by the model and thus eGFR measurement would be indicated, are referred to as true positives (TPs). Patients with an eGFR ≥60 ml/min/1.73 m^2^ or ≥45 ml/min/1.73 m^2^, who were not identified as being at risk, are referred to as true negatives (TNs). Patients with an eGFR <60 ml/min/1.73 m^2^ or <45 ml/min/1.73 m^2^, who were not detected by the model, are referred to as false negatives (FNs) and patients with eGFR ≥60 ml/min/1.73 m^2^ or ≥45 ml/min/1.73 m^2^, who were erroneously identified as being at risk by the model, are referred to as false positives (FPs).

### Sample size analysis

The prevalence of patients with an eGFR <60 ml/min/1.73 m^2^ in a previous study population was 11 % [26]. For model 3, a sensitivity of 89.3 % was obtained. We aimed to have a higher sensitivity with the same 95 % CI interval. Assuming a sensitivity of 90 % and similar 95 % CI, we had to include at least 82 patients with an eGFR <60 ml/min/1.73 m^2^. Based on the prevalence, 745 patients would be sufficient for model 3. For model 4, a sensitivity of 76.8 % was obtained in the previous study. We aimed to have a higher sensitivity with the same 95 % CI interval. Assuming a sensitivity of 80 % and similar 95 % CI, we had to include at least 86 patients with a eGFR <60 ml/min/1.73 m^2^. Based on the prevalence, 782 patients would be sufficient for model 4.

We included 1,000 patients, comparable to the number of patients included in a previous study [17].

### Statistical analysis

Normally distributed baseline data were presented as mean ± standard deviation (SD) and categorical baseline data were presented as numbers and proportions.

For both models, we firstly studied the association with the presence of kidney disease stage 3A or higher (eGFR <60 ml/min/1.73 m^2^) or 3B or higher (eGFR <45 ml/min/1.73 m^2^) by applying the χ^2^ test. For this aim, we constructed 2 × 2 data on eGFR <60 ml/min/1.73 m^2^ or <45 ml/min/1.73 m^2^ and the different screening models. We present sensitivity (TP/TP + FN) and false positive rates (FP/FP + TP) per model. The fit of the different models to the data was compared using the -2 log likelihood; a lower -2 log likelihood indicates a better fit.

We performed all statistical analyses using statistical analysis software (SPSS, version 22.0; IBM, Armonk, NY, USA). We considered a *p* value of <0.05 as statistically significant.

## Results

### Study design

**Fig. 1:**
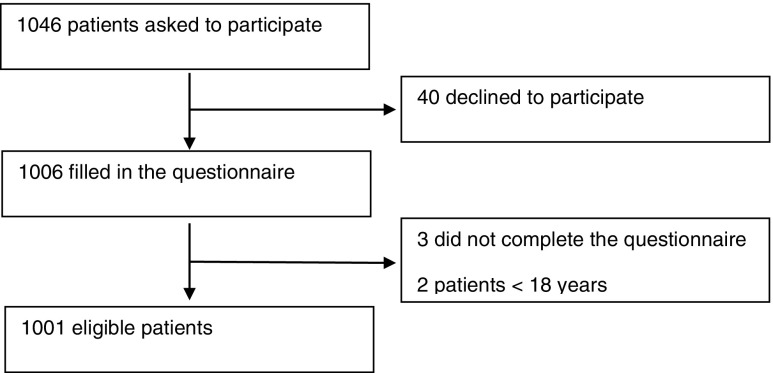
Flow diagram of participants

We approached 1,046 patients for the questionnaire. Of these patients 40 did not want to participate. Additionally, three patients did not fill out the form completely. Two patients aged <18 years were excluded. Finally, we included 1,001 patients for analysis, of which 985 (98.4 %) were outpatients and 16 (1.6 %) were patients admitted to the hospital. A flow diagram is shown in Fig. 1.

### Patient characteristics and kidney disease

We included 542 (55.1 %) men and 449 (44.9 %) women with a mean age of 60.36 years ± 13.13 years. The mean serum creatinine at baseline was 78.52 μmol/l ± 39.36 μmol/l.

We included 909 (90.8 %) patients with an eGFR ≥60 ml/mg/1.73 m^2^. There were 81 (8.1 %) patients with an eGFR of 45-59 ml/mg/1.73 m^2^ (stage 3A; mean eGFR of 54.21 ± 4.05 ml/min/1.73 m^2^), six (0.6 %) patients with an eGFR of 30-44 ml/min/1.73 m^2^ (stage 3B; mean eGFR of 38.17 ± 4.54 ml/min/1.73 m^2^) and five (0.5 %) patients with an eGFR of 15-29 ml/min/1.73 m^2^ (stage 4 and 5; mean eGFR of 15.00 ± 0.00 ml/min/1.73 m^2^). In total there were 92 (9.2 %) patients with an eGFR <60 ml/min/1.73 m^2^ and 11 (1.1 %) patients with an eGFR <45 ml/min/1.73 m^2^.

In half of the patients (509/1,001; 50.8 %), eGFR was measured less than 1 month before IV CECT and in 994 (99.3 %) patients within 12 months. *See* Table 1 for details on baseline characteristics and baseline eGFR measurements.

**Table 1:** Baseline characteristics of the included patients

	Total study population (*n* = 1,001)	eGFR ≥60 ml/min/1.73 m^2^ (*n* = 909)	eGFR 45-59 ml/min/1.73 m^2^ (*n* = 81)	eGFR 30-44 ml/min/1.73 m^2^ (*n* = 6)	eGFR 15-29 ml/min/1.73 m^2^ (*n* = 5)
Baseline characteristics					
Male, *n* (%)	552 (551.%)	501 (55.1 %)	45 (55.6 %)	3 (50.0 %)	3 (60.0 %)
Female, *n* (%)	449 (44.9 %)	408 (44.9 %)	36 (44.4 %)	3 (50.0 %)	2 (40.0 %)
Age (years) mean ±	60.36 ± 13.13/	59.86 ± 13.21/	65.64 ± 10.63/	67.83 ± 14.28/	55.40 ± 16.32/
SD/range	18-92	18-92	41-89	42-79	35-79
Afro European, *n* (%)	53 (5.3 %)	47 (5.1 %)	7 (8.6 %)	0 (0 %)	0 (0 %)
Length (cm) mean ± SD	173.94 ± 9.99	174.03 ± 10.09	172.90 ± 9.26	172.00 ± 3.63	175.40 ± 9.89
Weight (kg) mean ± SD	77. 29 ± 16.04	76.91 ± 15.88	81.63 ± 17.35	83.17 ± 13.47	69.60 ± 17.62
Kidney function					
Serum creatinine (μmol/ml) mean ± SD^a^	78.52 ± 39.36^a^	73.26 ± 14.29^a^	105.67 ± 14.69	140.83 ± 25.09	611.25 ± 160.43
eGFR (ml/min/1.73 m^2^) mean ± SD	-	-	54.21 ± 4.05	38.17±4.54	15 ± 0.00^c^
Interval between eGFR and CT exam^b^					
Within 1 month	509	451^a^	52	2	4
Between 1-3 months	264	241	19	4	0
Between 3-12 months	221	212	8	0	1
More than 12 months	6	4	2	0	0

^a^Missing one patient; serum creatinine was not mentioned

^b^Missing one patient with eGFR >60 ml/min/1.73 m^2^; it was not clear when eGFR was determined

^c^All patients had an eGFR of 15 ml/min/1.73 m^2^

### CT characteristics

Most CECT examinations were related to malignancy (*n* = 673, 67.3 %) and CT of the chest and abdomen was most frequent (*n* = 313, 31.3 %). Details on the type of scan and indication are presented in Table 2.

**Table 2:** Type of scan performed and the indication for patients undergoing intravenous contrast-enhanced CT

	Total study population (*n* = 1,001)	eGFR ≥60 ml/min/1.73 m^2^ (*n* = 909)	eGFR 45-59 ml/min/1.73 m^2^ (*n* = 81)	eGFR 30-44 ml/min/1.73 m^2^ (*n* = 6)	eGFR 15-29 ml/min/1.73 m^2^ (*n* = 5)
Type of CT scan					
Neck/chest/abdomen, *n* (%)	50 (5.0 %)	47 (5.2 %)	3 (3.7 %)	-	-
Chest/abdomen, *n* (%)	313 (31.3 %)	285 (31.4 %)	28 (34.6 %)	-	-
Chest, *n* (%)	103 (10.3 %)	93 (10.2 %)	8 (9.9 %)	2 (33.3 %)	-
Abdomen, *n* (%)	147 (14.7 %)	135 (14.9 %)	10 (12.3 %)	-	2 (40.0 %)
Pancreas, *n* (%)	69 (6.9 %)	63 (6.9 %)	5 (6.2 %)	1 (16.7 %)	-
Liver, *n* (%)	25 (2.5 %)	25 (2.8 %)	-	-	-
Kidney (or adrenal gland), *n* (%)	155 (15.5 %)	137 (15.1 %)	15 (18.5 %)	2 (33.3 %)	1 (10.0 %)
Oesophagus, *n* (%)	33 (3.3 %)	31 (3.4 %)	2 (2.5 %)	-	-
CT angiography (coronary arteries, aorta, peripheral vascular disease), *n* (%)	99 (9.9 %)	87 (9.6 %)	9 (11.1 %)	1 (16.7 %)	2 (40.0 %)
Other (skeletal/cerebrum), *n* (%)	7 (0.7 %)	6 (0.7 %)	1 (1.2 %)	-	-
Indication CT scan					
Malignancy, *n* (%)^a^	673 (67.2 %)	614 (67.5 %)	54 (66.7 %)	4 (66.7 %)	1 (20.0 %)
Vascular disease, *n* (%)^b^	90 (9.0 %)	77 (8.5 %)	10 (12.3 %)	1 (16.7 %)	2 (40.0 %)
Nephrological disease, *n* (%)^c^	44 (4.4 %)	40 (4.4 %)	4 (4.9 %)	-	-
Kidney donation, *n* (%)	23 (2.3 %)	22 (2.4 %)	1 (1.2 %)	-	-
Other, *n* (%)^d^	171 (17.1 %)	156 (17.2 %)	12 (14.8 %)	1 (16.7 %)	2 (40.0 %)

^a^Suspected, staging of malignancy or follow-up of malignancy

^b^Including cardiac disease, vascular deformation, pulmonary embolism, angina pectoris

^c^Including macroscopic haematuria

^d^Including cysts in liver, kidney, pancreas and infections

### Risk factors

The most prevalent risk factors were hypertension (*n* = 308, 30.8 %), cardiovascular disease (*n* = 212, 21.2 %) and a history of urological/nephrological disease (*n* = 187, 18.7 %). *See* Table 3 for details on risk factors.

**Table 3:** Distribution of risk factors in patients undergoing intravenous contrast-enhanced CT

	Total study population (*n* = 1,001)	eGFR ≥60 ml/min/1.73 m^2^ (*n* = 909)	eGFR 45-59 ml/min/1.73 m^2^ (*n* = 81)	eGFR 30-44 ml/min/1.73 m^2^ (*n* = 6)	eGFR 15-29 ml/min/1.73 m^2^ (*n* = 5)
Multiple myeloma or M. Waldenström, *n* (%)	6 (0.6 %)	6 (0.6)	-	-	-
Diabetes mellitus, *n* (%)	130 (13.0 %)	117 (12.9 %)	10 (12.3 %)	2 (33.3 %)	1 (20.0 %)
Hypertension, *n* (%)	308 (30.8 %)	255 (28.1 %)	43 (53.1 %)	6 (100.0 %)	4 (80.0 %)
Congestive heart failure	102 (10.2 %)	89 (9.8 %)	11 (13.6 %)	0 (0.0 %)	2 (40.0 %)
Other cardiovascular disease	137 (13.7 %)	121 (13.3 %)	12 (14.8 %)	3 (50.0 %)	1 (20.0 %)
Cardiovascular disease, *n* (%)^a^	212 (21.2 %)	187 (20.6 %)	20 (24.7 %)	3 (50.0 %)	2 (40.0 %)
Anaemia, *n* (%)	84 (8.4 %)	76 (8.4 %)	4 (4.9 %)	1 (16.7 %)	3 (60.0 %)
Urological disease, *n* (%)^b^	187 (18.7 %)	132 (14.5 %)	45 (55.6 %)	5 (83.3 %)	5 (100.0 %)
Age >75 years, *n* (%)	120 (12.0 %)	101 (11.1 %)	15 (18.5 %)	3 (50 %)	1 (20.0 %)

^a^Combined cardiovascular disease: congestive heart disease or other cardiovascular disease, such as peripheral arterial disease, stroke, etc

^b^Including urological/nephrological history and known decreased kidney function

### Combinations of risk factors

#### Model A

A total of 543 patients were detected who had diabetes mellitus, history of urological/nephrological disease, cardiovascular disease (all cardiovascular disease including congestive heart failure and peripheral arterial disease) and hypertension.

##### eGFR <60 ml/min/1.73 m^2^

There was a significant association between the risk factors for kidney disease in this model and eGFR <60 ml/min/1.73 m^2^ (*p* < 0.0001; -2 log likelihood, 561.3). The model detected 462 patients with an eGFR ≥60 ml/min/1.73 m^2^ (FP) and 81 with an eGFR <60 ml/min/1.73 m^2^ (TP). There were 11 FNs. Of the 11 FNs, seven had no risk factors. Of the remaining patients, three were >75 years of age and another patient was anaemic. These last four patients had only one risk factor, indicating there was no increased chance of CIN after contrast medium administration. *See* Table 4.

**Table 4:** Screening strategies for eGFR <60 ml/min/1.73 m^2^

Risk models for screening for patients with eGFR <60 ml/min/1.73 m^2^		Patients with eGFR <60 ml/min/1.73 m^2^ (*n* = 91)		Patients with eGFR ≥60 ml/min/1.73 m^2^ (*n* = 909)		χ^2^ test	Sensitivity (95 % CI)	False positive rate (95 % CI)
Models^a^	-2 log likelihood	True positive (TP), *n*	False negative (FN), *n*	False positive (FP), *n*	True negative (TN), *n*	*p* values	TP (TP + FN)	FP/(TP + FP)
Model A (*n* = 543)	561.3	81	11	462	448	<0.001	88.0 % (81.4-94.7 %)	85.1 % (82.1-88.1 %)
Model B (*n* = 420)	556.7	70	22	350	560	<0.001	76.1 % (67.4-84.8 %)	83.3 % (79.8-86.9 %)
Overlap^b^		67	8	319	406			

^a^The model with the lowest -2 log likelihood indicates a better fit. Model A includes diabetes mellitus, history of urological/nephrological disease, cardiovascular disease (all cardiovascular disease including congestive heart failure, and peripheral arterial disease) and hypertension. Model B includes diabetes mellitus, history of urological/nephrological disease, age >75 years and congestive heart failure

^b^Number of overlaps between models A and B

##### eGFR <45 ml/min/1.73 m^2^

This model showed significant association between risk factors for kidney disease and eGFR <45 ml/min/1.73 m^2^ (*p* < 0.002; -2 log likelihood, 107.6). This model detected 532 patients with an eGFR ≥45 ml/min/1.73 m^2^ (FP), all 11 with an eGFR <45 ml/min/1.73 m^2^ (TP) were detected. *See* Table 5.

**Table 5:** Screening strategies for eGFR <45 ml/min/1.73 m^2^

Risk models for screening for patients with eGFR <45 ml/min/1.73 m^2^		Patients with eGFR <45 ml/min/1.73 m^2^ (*n* = 11)		Patients with eGFR ≥45 ml/min/1.73 m^2^ (*n* = 990)		χ^2^ test	Sensitivity (95 % CI)	False positive rate (95 % CI)
Models^a^	-2 log likelihood	True positive (TP), *n*	False negative (FN), *n*	False positive (FP), *n*	True negative (TN), *n*	*p* values	TP (TP + FN)	FP/(TP + FP)
Model A (*n* = 543)	107.6	11	0	532	458	0.002	100.0 % (NA)	98.0 % (96.8-99.2 %
Model B (*n* = 420)	101.8	11	0	409	581	<0.001	100.0 % (NA)	97.4 % (95.9-98.9 %)
Overlap^b^		11	0	365	414			

^a^The model with the lowest -2 log likelihood indicates a better fit. Model A includes diabetes mellitus, history of urological/nephrological disease, cardiovascular disease (all cardiovascular disease including congestive heart failure, and peripheral arterial disease) and hypertension

Model B includes diabetes mellitus, history of urological/nephrological disease, age >75 years and congestive heart failure

^b^Number of overlaps between models A and B

#### Model B

A total of 420 patients were detected who had diabetes mellitus, history of urological/nephrological disease, age >75 years and congestive heart failure.

##### eGFR <60 ml/min/1.73 m^2^

In this model there was significant association between the risk factors for kidney disease and eGFR <60 ml/min/1.73 m^2^ (*p* < 0.0001; -2 log likelihood, 565.7). The model detected 350 patients with eGFR ≥60 ml/min/1.73 m^2^ (FP) and 70 with an eGFR <60 ml/min/1.73 m^2^ (TP). There were 22 FNs. Of the 22 FN patients, seven patients did not have any risk factors for kidney disease. Of the 15 remaining patients, 12 patients had only one risk factor (11 hypertension and one anaemia). Three patients had two risk factors (hypertension and peripheral disease). *See* Table 4.

##### eGFR <45 ml/min/1.73 m^2^

There was significant association between the risk factors and eGFR <45 ml/min/1.73 m^2^ (*p* < 0.0001; -2 log likelihood, 101.8). The model detected 409 patients with an eGFR ≥45 ml/min/1.73 m^2^ (FP) and all 11 patients with an eGFR <45 ml/min/1.73 m^2^ (TP). *See* Table 5.

## Discussion

This study shows that the number of eGFR measurements to detect pre-existent kidney disease can be safely reduced in patients undergoing IV CECT. This can be achieved by first identifying patients at risk of CIN by monitoring four objective risk factors and determining eGFR solely in those patients with one of these risk factors. Screening with simplified models may save time and eventually may save costs of superfluous eGFR measurements.

If we use a model (model A) including diabetes mellitus, history of urological/nephrological disease, cardiovascular disease (including congestive heart failure) and hypertension, there is a reduction of 46 % eGFR measurements (from 1,001 to 543). If we use model B, including diabetes mellitus, history of urological/nephrological disease, age >75 years and congestive heart failure, we would achieve a reduction in eGFR measurements of 58 % (from 1,001 to 420 patients). This is in accordance with the previous study of Moos et al. [26], where reductions of eGFR measurements were 38 % and 56 % respectively.

Statistically, model B provides the best fit. The reduction of FPs in model A is less substantial and the -2 likelihood also suggests a lesser fit of this model compared with model B. However, model A resulted in less FNs compared with model B when a cut-off value of eGFR <60 ml/min/1.73 m^2^ was considered (11 and 22 respectively). As stated before, two European guidelines recently redefined their risk profile for patients receiving intravenous iodinated contrast medium. Patients at risk for CIN are defined as patients with pre-existent eGFR of <40 ml/min/1.73 m^2^ or <45 ml/min/1.73 m^2^ [15,21]. By using an eGFR cut-off value of <45 ml/min/1.73 m^2^ both models have a sensitivity of 100 % (all patients with an eGFR of <45 ml/min/1.73 m^2^ were detected). This is similar to the findings in the study of Moos et al. [26].

A survey among European radiologists performed by Reddan et al. [30] showed a highly variable insight in the definition, impact and risk factors for CIN exists among European radiologists. While there was good awareness amongst the radiologists, at least 10 % of respondents did not identify dehydration, diabetes mellitus or nephrotoxic medication as risk factors. This might be a result of the extensive list of risk factors for decreased kidney function described in CIN prevention guidelines that might be cumbersome to apply in daily practice.

A study by Choyke et al. [31] showed that when patients are asked to fill out a simple questionnaire before IV CECT, most patients with normal creatinine levels can be identified and this could reduce the number of superfluous pre-procedural eGFR measurements by two-thirds. These investigators determined that if patients stated not to have any risk factors for kidney disease, 99 % of these patients had no pre-existent kidney disease.

Another study by Lui et al. showed that even in patients aged >60 years of age, in 98 % of the cases serum creatinine levels were normal when patients indicated not to have any risk factors. This indicates that the specificity of these questionnaires is 98-99 % [32]. However, data on number of patients with one or more risk factors and data on numbers on patients with kidney disease were missing.

If we combine our findings with this recent literature, it can be stated that by presenting patients a simple questionnaire including four risk factors before IV CECT, superfluous eGFR measurements could be substantially reduced without missing patients with an eGFR <45 ml/min/1.73 m^2^.

### Limitations

Our study has some limitations. As the patient information was collected by a questionnaire and no hospital electronic patient record was consulted for risk factors, relevant information could be missed. However, the questions are objective parameters, which can be easily filled in by the patients. The risk factors are often not systematically entered in medical records. Extracting these data from medical records would be time-consuming, while our goal is to reduce work-load and costs. In routine practice the physician is asked to fill in a CT referral form with eGFR and risk factors in case of an eGFR <60 ml/min/1.73 m^2^. In an interim analysis of these patients (500 patients), a total of three patients were identified as high-risk for developing CIN based on eGFR 45-59 ml/min/1.73 m^2^ and diabetes mellitus. A total of 17 patients were identified as high-risk based on eGFR 45-59 ml/min/1.73 m^2^ and ≥2 other risk factors (not diabetes mellitus). The referring physician only identified one of these high-risk patients in both of these groups and did not mention the risk factor diabetes mellitus in two patients. Due to these discrepancies, we only used the questionnaire data. The results of this study are in accordance to a previous study in which complete data were collected, so we assume no critical information was missed [26].

Another limitation is that we did not include all patients undergoing IV CECT during our inclusion period. The patients were randomly asked at the desk to fill in the questionnaire, depending on whether there was enough time for patients to fill in the questionnaire. No other selection criteria were defined.

Another limitation is that in this study a comparable patient population was included as in the previously reported study [26]. However, in the previous study the prevalence of eGFR <45 ml/min/1.73 m^2^ was 30/1001 = 3 % [26]. Although this prevalence was three times higher, the same results were found. Therefore we believe that these data can be generalised in a patient population undergoing IV CECT.

Because this study population was undergoing IV CECT, the likelihood of this population being ill and having more risk factors is probably higher than in a general population. Therefore validation of the model in populations with a different spectrum is mandatory.

## Conclusions

In this study we validated the models developed in a previous study. The model including a combination of diabetes mellitus, history of urological/nephrological disease, age >75 years and congestive heart failure for detecting pre-existent kidney disease prior to intravenous contrast medium administration is safe and can lead to a large reduction in superfluous eGFR measurements and presumably also a substantial reduction of costs.

## Electronic supplementary material

Below is the link to the electronic supplementary material.

ESM 1 (DOC 41 kb)
