# Supplementary material for: Prediction of presence of kidney disease in patients undergoing intravenous iodinated contrast enhanced computed tomography: a validation study
Source: Eur Radiol. 2016 Jul 19;27(4):1613–21. doi: 10.1007/s00330-016-4478-0 (PMC5334394; doi:10.1007/s00330-016-4478-0)
Supplement: Supplementary file 1 — (DOC 41 kb) [file 330_2016_4478_MOESM1_ESM.doc]

**Date:.…………………………………………………….**

**Name:……………………………………………………**

**Patient ID:.………………………………………………**

|  |  |
| --- | --- |

**DEMOGRAFIC DATA**

The following questions are about your age, length and weight. Please fill in the details without decimals, so rounded numbers.

**1.1.** Gender *(please check box that applies)*:

O Male

O Female

**1.2.** Age:……….years *(whole years)*

**1.3.** Length:…………cm *(e.g. 1.80 meter = 180 cm)*

**1.4.** Weight:……….kg *(whole kilograms)*

**RISK FACTORS**

The following question is about medical conditions, for which you require medicine and/or medical treatment. *Please check the box ‘Yes’ if applicable and ‘No’ if not applicable.*

**2.1.** Do you have (had) one of these medical conditions?

| **Medical conditions** | | **Yes** | **No** |
| --- | --- | --- | --- |
| 1. | Kahler’s disease (= Multiple Myeloma) or Waldenström’s disease | O | O |
| 2 | Diabetes | O | O |
| 3. | Hypertension | O | O |
| 4. | Heart failure | O | O |
| 5. | Other cardiac or vascular diseases (e.g. stroke, claudication etc.) | O | O |
| 6. | Anaemia | O | O |
| 7. | Any disease of kidneys or urinary tract (in the past) | O | O |
| 8. | Renal impairment or renal failure | O | O |

***-End of the questionnaire-***

**Thank you for your help!**
